# Inhibiting receptor tyrosine kinase AXL with small molecule inhibitor BMS-777607 reduces glioblastoma growth, migration, and invasion *in vitro* and *in vivo*

**DOI:** 10.18632/oncotarget.7130

**Published:** 2016-02-02

**Authors:** Julia Onken, Robert Torka, Sören Korsing, Josefine Radke, Irina Krementeskaia, Melina Nieminen, Xi Bai, Axel Ullrich, Frank Heppner, Peter Vajkoczy

**Affiliations:** ^1^ Department of Neurosurgery, Charité, Berlin, Germany; ^2^ Institute of Neuropathology, Charité, Berlin, Germany; ^3^ Department of Molecular Biology, Max-Planck Institute of Biochemistry, Martinsried, Germany

**Keywords:** glioblastoma multiforme (GBM), small molecule inhibitor BMS-777607, receptor tyrosine kinase AXL (RTK-AXL), invasion, xenograft model

## Abstract

**Purpose:**

Receptor tyrosine kinase AXL (RTK-AXL) is regarded as suitable target in glioma therapy. Here we evaluate the anti-tumoral effect of small molecule inhibitor BMS-777607 targeting RTK-AXL in a preclinical glioma model and provide evidence that RTK-AXL is expressed and phosphorylated in primary and recurrent glioblastoma multiforme (GBM).

**Experimental design:**

We studied the impact of BMS-777607 targeting RTK-AXL in GBM models *in vitro* and *in vivo* utilizing glioma cells SF126 and U118MG. Impact on proliferation, apoptosis and angiogenesis was investigated by immunohistochemistry (IHC) and functional assays *in vitro* and *in vivo*. Tumor growth was assessed with MRI. Human GBM tissue was analyzed in terms of RTK-AXL phosphorylation by immunoprecipitation and immunohistochemistry.

**Results:**

BMS-777607 displayed various anti-cancer effects dependent on increased apoptosis, decreased proliferation and migration *in vitro* and *ex vivo* in SF126 and U118 GBM cells. *In vivo* we observed a 56% tumor volume reduction in SF126 xenografts and remission in U118MG xenografts of more than 91%. The tube formation assay confirmed the anti-angiogenic effect of BMS-777607, which became also apparent in tumor xenografts. IHC of human GBM tissue localized phosphorylated RTK-AXL in hypercellular tumor regions, the migratory front of tumor cells in pseudo-palisades, and in vascular proliferates within the tumor. We further proved RTK-AXL phosphorylation in primary and recurrent disease state.

**Conclusion:**

Collectively, these data strongly suggest that targeting RTK-AXL with BMS-777607 could represent a novel and potent regimen for the treatment of primary and recurrent GBM.

## INTRODUCTION

Malignant gliomas are the most common and most aggressive brain tumors due to their highly invasive growth pattern, proliferative capacities and heterogeneity [1]. Despite, multimodal aggressive therapy with chemotherapy, radiation, and surgery, less than 10% of patients with the diagnosis glioblastoma multiforme (GBM) survive 5 years beyond diagnosis [2, 3]. For the development of new therapeutic strategies it is unavoidable to understand the mechanisms of gliomagenesis and malignancy criteria of GBMs. Current investigations aim to uncover novel therapeutic approaches by exploring the oncogenic mechanisms and unique potential targets of GBMs [4].

The receptor tyrosine kinase AXL (RTK-AXL) displays a new promising target in glioma therapy [5]. RTK-AXL is characterized by an extracellular domain consisting of two immunoglobulin-like domains in juxtaposition of two fibronectin type III domains, typical for cell adhesion molecules of the immunoglobulin superfamily [6]. The growth arrest–specific gene 6 (Gas6) is the natural ligand of RTK-AXL. RTK-AXL/Gas6 signaling is in charge of regulating survival, proliferation, and migration in different types of cells *in vitro*, including tumor-derived epithelial, mesenchymal, and hematopoietic cell lines [6–8]. RTK-AXL/Gas6 overexpression has been described in a multitude of human cancers, including GBM, colon, breast, prostate, thyroid, lung cancer, and malignant melanoma [6, 7, 9]. It has been shown that overexpression of RTK-AXL and Gas6 in GBM tissue is associated with reduced time to progression and overall survival in these patients [10, 11]. Its oncogenic effect is explained by Gas6-dependent signaling through RTK-AXL resulting in phosphorylation of AKT and ERK 1/2 [12]. Furthermore, anti-apoptotic Bcl-2 family members (e.g., Bcl-2) are unregulated while pro-apoptotic family members (e.g., BAD) are inactivated [7, 13, 14].

Its exceptional role in GBM was presented in previous studies showing that experimental inhibition of the RTK-AXL pathway with dominant negative-mutant glioma cells of AXL receptor (SF126 AXL-DN) suppresses glioma growth and prolongs survival in orthotopic tumor model in mice [15]. SF126 AXL-DN cells display an attenuated locomotor or migration activity with reduced formation of filopodia and loss of cell-to-cell interaction. Tumor cell motility is impaired in SF126 AXL-DN cells, which indicates that these cells are unable to invade normal brain tissue. Previous results point to the fact that specific targeting of RTK-AXL supposes a promising approach to intervene GBM progression [16, 17].

Therefore, we studied a targeted inhibition of RTK-AXL phosphorylation with selective small molecule inhibitor BMS-777607 *in vitro, ex vivo*, and *in vivo*. We observed significant regression of intracranial tumors due to treatment with BMS-777607. These effects were mediated through increased apoptosis, reduced proliferation, migration, and neoangiogenesis. According to the experimental results, we are the first to prove, that RTK-AXL is phosphorylated in human GBM tissue and that it is expressed in proliferating cells, in the migratory front of tumor cells, and in vascular proliferates. Additionally, we demonstrate that RTK-AXL is strongly phosphorylated in both primary and recurrent GBMs.

## RESULTS

### BMS-777607 selectively blocks AXL phosphorylation in U118MG and SF126 cells *in vitro* and *in vivo*

**Figure 1:**
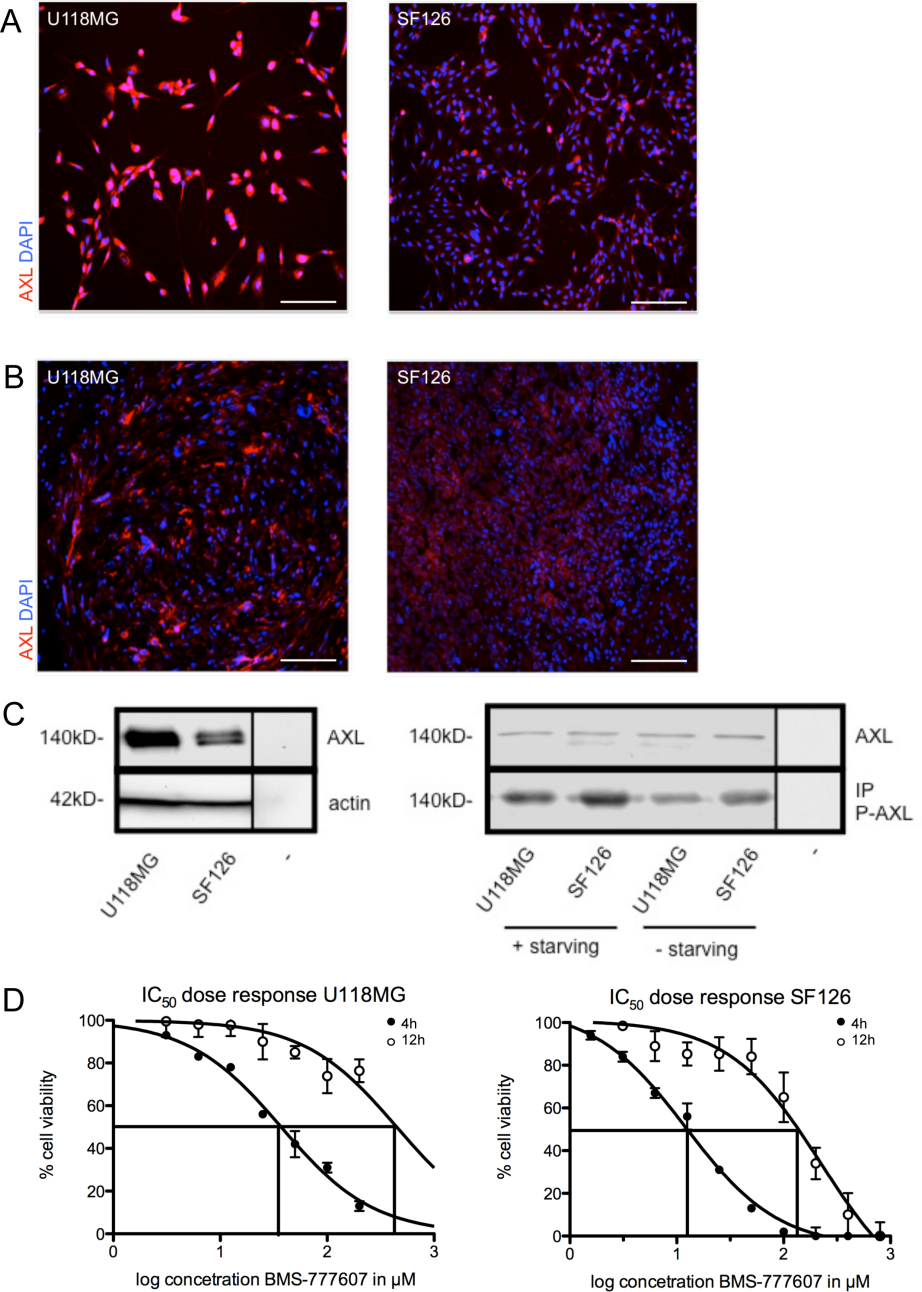
(**A**) RTK-AXL expression of U118MG and SF126 cells *in vitro*. (**B**) RTK-AXL expression of U118MG and SF126 xenografts *in vivo*. Scale bar indicates 50 μm. (**C**) left image: Western blot showing base line expression of RTK-AXL in cell lysates of U118MG and SF126 (− = negative control). (C) right image: Western blot analysis of changes in the phosphorylation of RTK-AXL under starving conditions compared to standard culture conditions (− = negative control). (**D**) IC_50_ of U118MG and SF126 cells after treatment with BMS-777607 for 4 and 12 hours.

The expression of RTK-AXL in U118MG and SF126 cells *in vitro* and *in vivo* was confirmed by immunofluorescence staining of adherent cells (Figure 1A) and intracranial tumor tissue (Figure 1B). Western blot revealed a higher base line expression of RTK-AXL in U118MG compared to SF126 (Figure 1C, left image). We examined the phosphorylation of RTK-AXL under serum starved and standard conditions. We detected an increase of P-AXL in cells cultured under starving conditions (DMEM without FCS) compared to individual cell line cultured with DMEM containing 10% FCS (Figure 1C, right image).

**Figure 2:**
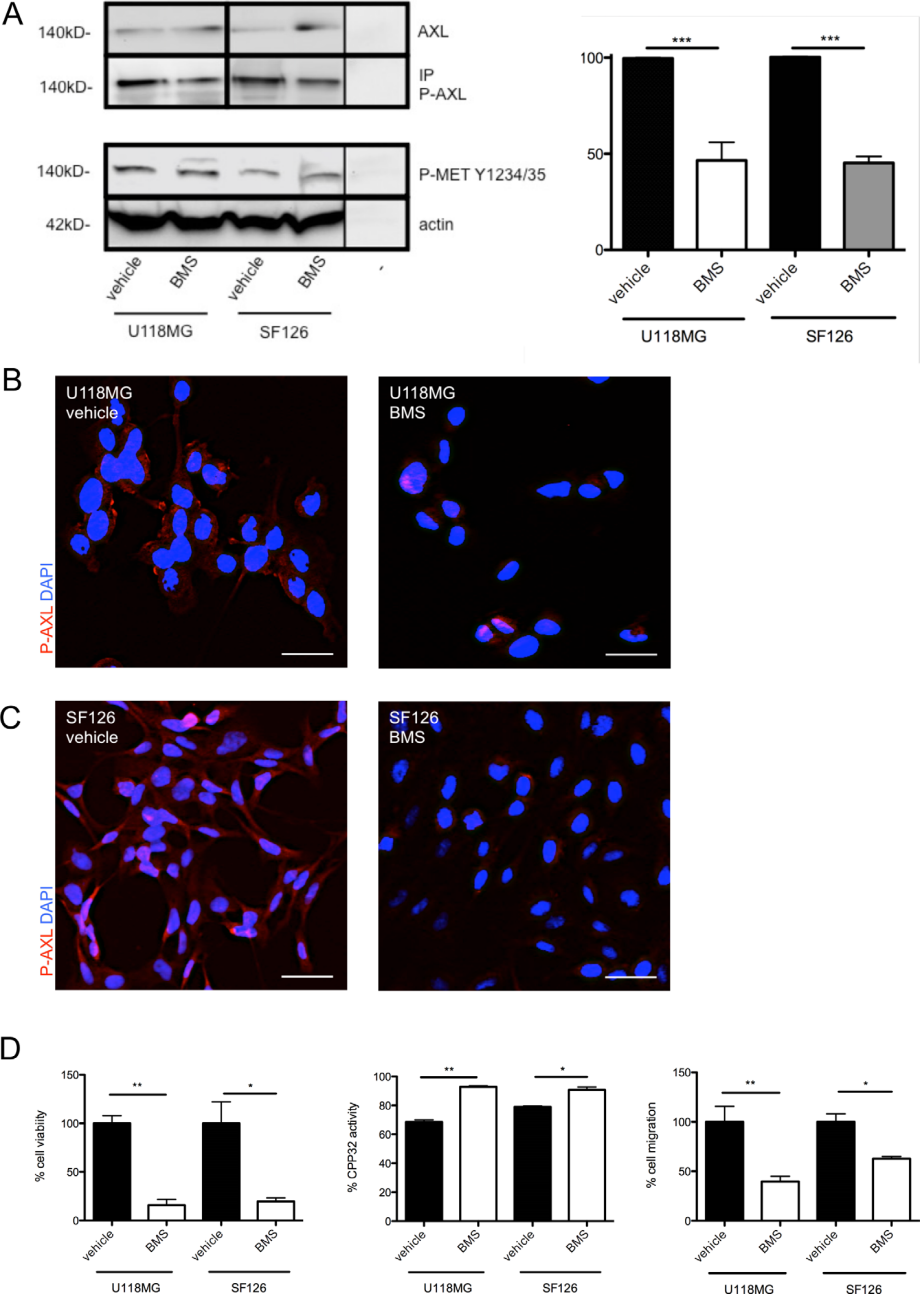
(**A**) left image: Western blot with IP of phosphorylated RTK-AXL and MET-kinase with cell lysates after 12 hours treatment with 12.5 μM BMS-777607 (− = negative control). (**A**) right image: Statistical analysis of P-AXL expression of three independent replicates (*n* = 3, ***p < 0.0001). (**B**) and (**C**) IHC of P-AXL in U118MG and SF126 after treatment with vehicle and BMS-777607. (**D**) left image: MTT results after repeated treatment with 12.5 μM BMS-777607 every 12 hours (*n* = 5, **p = 0.0011, *p = 0.025). (D) middle image: Results of apoptosis assay 24 hours after single treatment with 12.5 μM BMS-777607 (*n* = 3, **p = 0.0045, *p = 0.0289). (D) right image: Boyden chamber migration assay after 3 hours migration time under treatment with 12.5 μM BMS-777607 (*n* = 5, **p = 0.0045, *p = 0.0228).

The IC_50_ value of BMS-777607 was determined for U118MG and SF126 at 4 and 12 hours after single treatment (Figure 1D). Specific inhibition of phosphorylation of RTK-AXL by BMS-777607 was confirmed in western blot analysis. Following 12 hours of incubation, P-AXL was significantly reduced in both cell lines. At the same time phosphorylation of RTK-MET was unaffected confirming the selectivity of BMS-777607 against RTK-AXL at a dosage of 12.5 μM (Figure 2A). We therefore addressed further results as RTK-AXL dependent. Cell staining with anti-phospho-AXL antibody revealed membrane bound staining in both cell lines, which was reduced following incubation with the small molecule inhibitor of RTK-AXL BMS-777607 (Figure 2B, 2C).

### BMS-777607 decreases glioma cell viability and induces glioma cell apoptosis *in vitro*

The exposure of U118MG and SF126 glioma cells with 12.5 μM BMS-777607 resulted in significantly reduced cell numbers in MTT assay after 24 hours of treatment (Figure 2D, left image). Single application of 12.5 μM BMS-777607 led to a significant increase of CPP32 activity in U118MG and SF126 cell lines after 24 hours (Figure 2D, middle image). We concluded that reduced cell viability by BMS-777607 resulted from the induction of apoptosis and reduction of proliferation.

### BMS-777607 blocks glioma cell migration and invasive growth pattern

**Figure 3:**
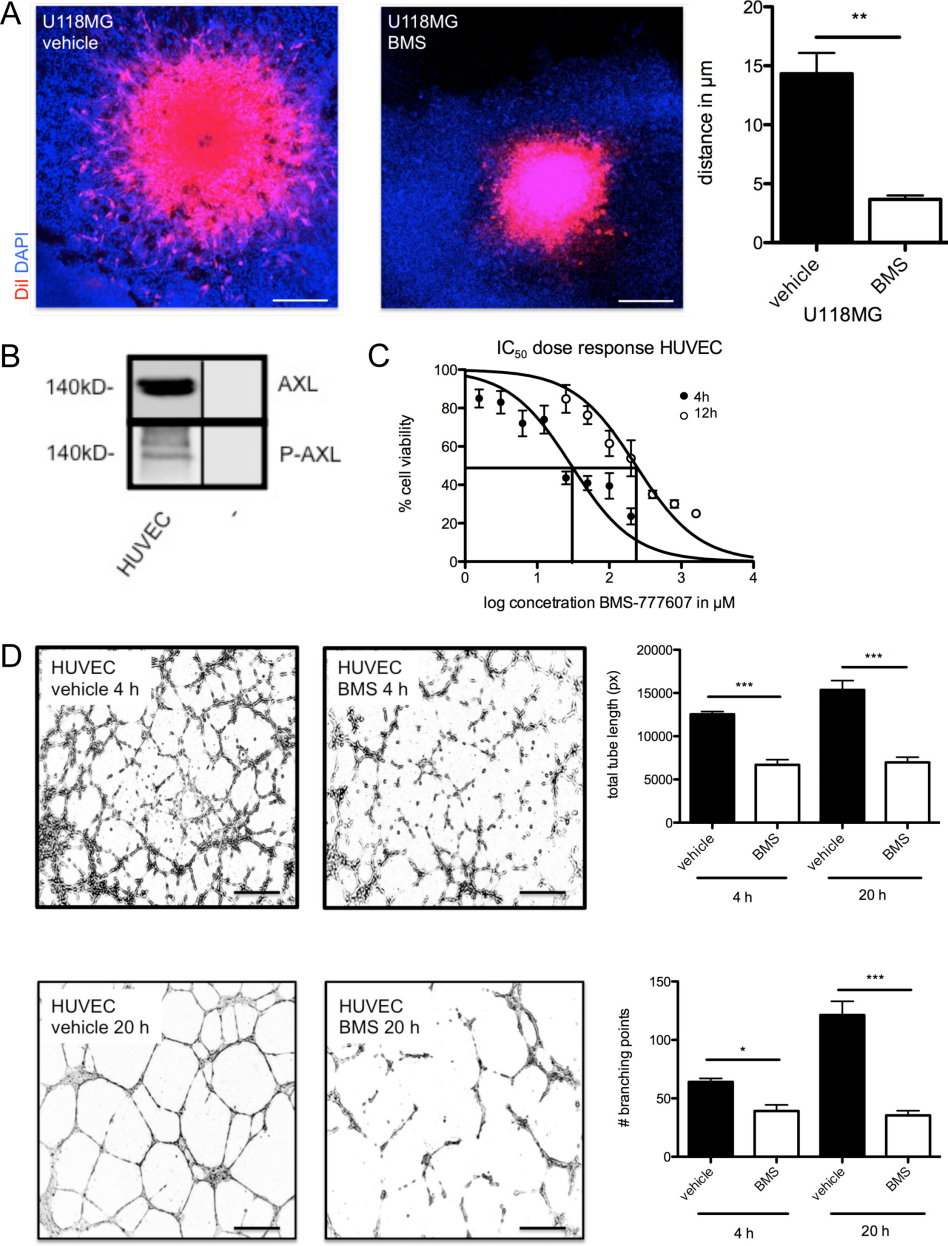
(**A**) Orthotopic brain slice invasion assay with implanted, DiI stained tumor cell spheroids of cell line U118MG. Images show data at day 8 under daily administration of BMS-777607 with corresponding statistical analysis (*n* = 8, **p = 0.0040). Results were obtained with confocal microscopy. Scale bar indicates 10 μm. (**B**) Expression of AXl and P-AXL in HUVECs (− = negative control). (**C**) IC_50_ value of BMS-777607 treatment in HUVECs. (**D**) left images: Tube formation assay with HUVECs after 4 and 20 hours and single administration of 12.5 μM BMS-777607. (D) right images: Statistical analysis at 4 and 20 hours of tube length (*n* = 5, 4 h: ***p < 0.0001, 20 h: ***p < 0.0001) and branching points (*n* = 5, 4 h: *p = 0.035, 20 h: ***p < 0.0001).

Next, we addressed the effects of BMS-777607 on glioma cell migration and invasion. Blockage of RTK-AXL signaling with initial dose of 12.5 μM BMS-777607 resulted in a significant decrease of migration rate in U118MG and SF126 cells measured by Boyden Chamber Migration assay after 3 hours (Figure 2D, left image). Invasive growth pattern of glioma cells was studied in the orthotropic brain slice culture model, a three-dimensional invasion assay closely related to an *in vivo* situation as the structure and organization of the brain tissue are preserved [19]. Under control conditions, only U118MG cells, but not SF126 cells, demonstrated invasive growth in this assay. Inhibition of RTK-AXL signaling with daily administration of 12.5 μM BMS-777607 abrogated tumor cell invasion into the brain tissue (Figure 3A, right image). Quantitative analysis using confocal microscopy confirmed this significant anti-invasive effect of BMS-777607 in this *ex vivo* model on day 8 (Figure 3A, left image).

### RTK-AXL is phosphorylated in HUVECs and inhibition displays antiangiogenic effect

RTK-AXL signaling has been also shown to be involved in endothelial cell proliferation and angiogenesis (5). Thus, we additionally studied the effects of BMS-777607 on endothelial cell biology using *in vitro* assays with HUVECs. Figure 3B shows RTK-AXL expression and RTK-AXL phosphorylation in HUVECs. The IC_50_ value of BMS-777607 was assessed as shown in Figure 3C. In accordance to this result, tube formation assay was carried out with 12.5 μM BMS-777607.

Consistent with *in vivo* findings we proved direct effect of BMS-777607 on endothelial cells HUVEC in tube formation assay. We observed a significant decrease of tube formation and branching points after 4 hours, which was still persistent after single treatment at the 20 hours time point (Figure 3D).

### I.p. administration of BMS-777607 selectively blocks RTK-AXL signaling *in vivo* and inhibits intracranial tumor growth in mouse xenografts

For *in vivo* experiments, U118MG and SF126 cells were implanted stereotactically into the brains of CD1NuNu mice. While U118MG xenografts showed a more invasive growth pattern, SF126 xenografts developed vascular proliferates, central necrosis and had a stronger proliferation rate (Supplementary Figure S2).

**Figure 4:**
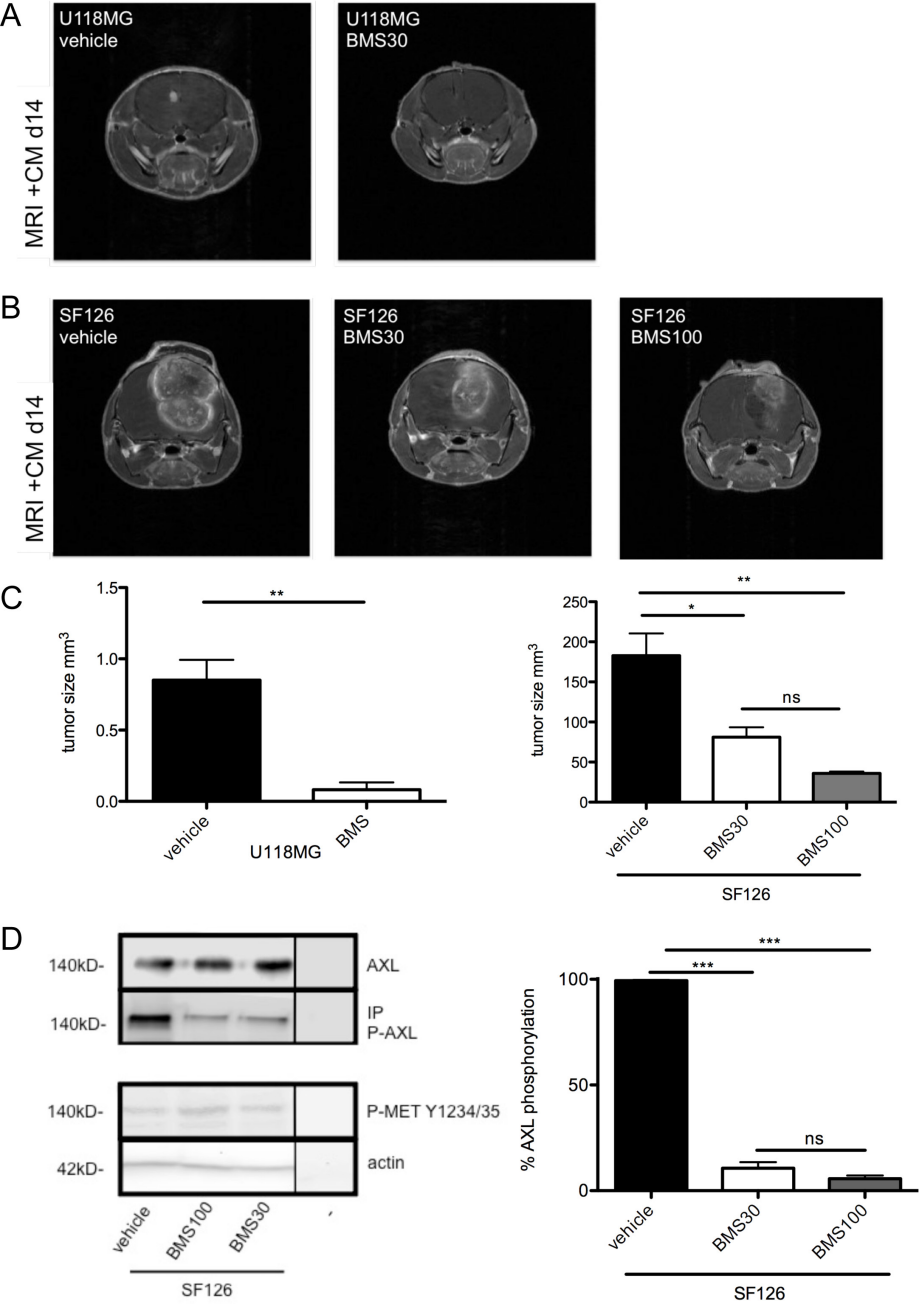
(**A**) MRI results of U118MG after 7 days of treatment with 30 mg/kg BW BMS-777607 (BMS30, *n* = 7) and vehicle at day 14 (*n* = 7). (**B**) MRI results of SF126 after 11 days of treatment with 30 mg/kg BW (BMS30, *n* = 5), 100 mg/kg BW BMS-777607 (BMS100, *n* = 7), and vehicle (*n* = 7) at day 14. MRI images are contrast enhanced. (**C**) Both images display tumor volumetric analysis after 14 days experiment. U118MG vehicle vs. BMS30 **p = 0.0012. SF126 vehicle vs. BMS30: *p = 0.015, vehicle vs. BMS100: **p = 0.0013, BMS30 vs. BMS100: ns. (**D**) left image: Western blot analysis with regulation of RTK-AXL and MET kinase phosphorylation on intracranial tumor tissue after i.p. administration of BMS-777607 at day 14 (− = negative control). (**D**) right image: Statistical analysis of three independent experiments of Western blot with tumor lysates (*n* = 3, ***p < 0.0001).

In both tumor models, treatment was initiated after proven tumor manifestation using MRI. In the more aggressive tumor SF126 model, treatment was started at day 3 after implantation while treatment in the U118MG model was started on day 7 after implantation (Supplementary Figure S3). In both models, BMS-777607 treatment (i.p. 2x/day) resulted in a significant decrease of intracranial tumor growth on day 14 after implantation. The most significant anti-tumor effect was seen in U118MG tumor model (Figure 4A). Here, 30 mg/kg BW BMS-777607 resulted in more than 90% tumor reduction after 6 days of treatment, in two cases we even observed complete tumor regression (*n* = 5, Figure 4C, left image). In the SF126 tumor model we detected a 56% tumor volume reduction. Increase of dosage up to 100 mg/kg BW lead to further regression. We showed that BMS-777607 displayed a dose dependent effect when applying it at 30 mg/kg BW and 100 mg/kg BW (Figure 4C, right image).

Protein analysis of the intracranial tumors revealed reduction of RTK-AXL phosphorylation on Western blot analysis. Furthermore, we did not observe compensatory MET kinase activation after 14 days of treatment (Figure 4D).

### BMS-777607 exerts multiple anti-tumor effects *in vivo*

**Figure 5:**
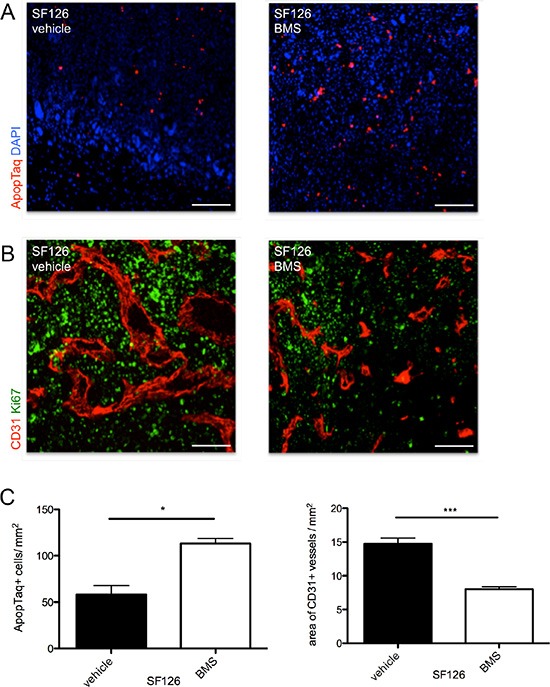
(**A**) Intratumoral apoptotic events in SF126 xenografts under treatment with BMS-777607 and vehicle. (**B**) CD31 staining (red) shows differences in vessel size and density under treatment with BMS-777607 *in vivo*. Proliferative activity is displayed qualitatively with Ki67 staining (green). (**C**) Images show corresponding statistical analysis of apoptotic events within tumor tissue (*n* = 3, *p = 0.0121) and statistical analysis of intratumoral vessel density (*n* = 3, ***p < 0.0001). Scale bar indicates 50 μm.

The treatment with BMS-777607 revealed multiple anti-tumor effects *in vivo*. In accordance with our *in vitro* data, BMS-777607 treatment resulted in a reduction of tumor cell proliferation and increase of apoptotic events in SF126 tumor xenografts compared to the control (*p* = 0.012, Figure 5A and Figure 5C, left image). Qualitative analysis of Ki67 expression within the tumor tissue showed a decreased proliferative activity under treatment with BMS-777607 (Figure 5B).

Having shown that inhibition of RTK-AXL phosphorylation in endothelial cells also resulted in an impaired tube formation, we also investigated the effect of BMS-777607 treatment on the glioma blood vessel surface and vascular architecture using immunohistochemistry. BMS-777607 reduced glioma vessel surface and vascular size, revealing its anti-angiogenic efficacy (Figure 5B and Figure 5C, right image). These findings were consistent in the border zone and center of the tumor where different angiogenic activities are usually observed (data not shown).

### Phosphorylated RTK-AXL is abundantly present in both primary and recurrent human GBM specimens

**Figure 6:**
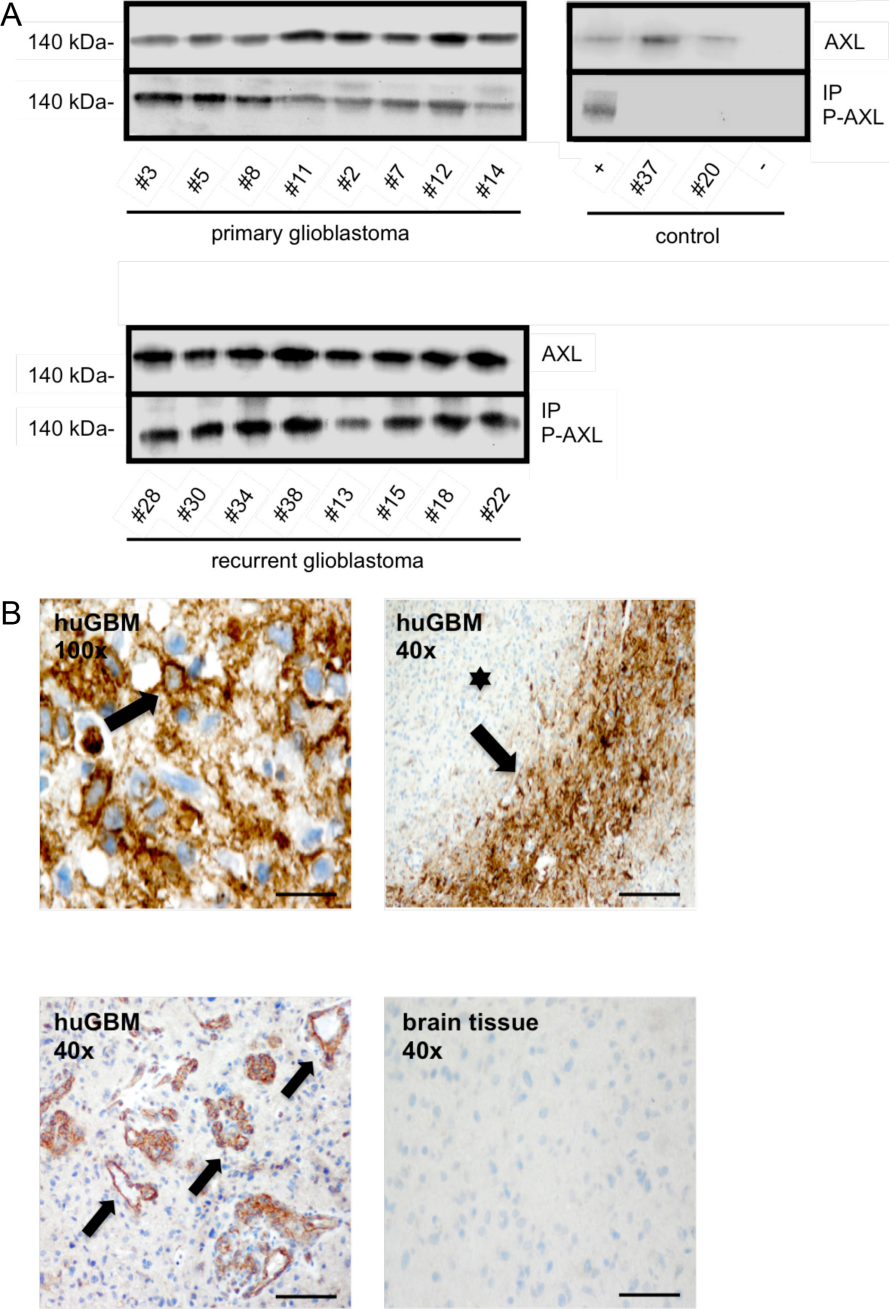
(**A**) Upper left blot shows IP of phosphorylated RTK-AXL in 8 patients with primary glioblastoma multiforme (pGBM), lower blot shows phosphorylated RTK-AXL in 8 patients with recurrent glioblastoma multiforme (rGBM). Upper right blot shows following control samples: positive ctrl: SF126 tumor mice, patient #37: meningeoma WHO°I, patient #20: arachnoidal cyst, negative ctrl: beads incubated with antibody and sample diluent. (**B**) Staining of phosphorylated RTK-AXL in human glioblastoma multiforme tissue (huGBM). Upper left image shows membrane bound staining of tumor cells (arrow). Scale bar of upper left image indicates 10 μm. Lower left image shows staining of phosphorylated RTK-AXL of tumor vessels (arrow). RTK-AXL phosphorylation is also observed in tumor pseudo-palisades (arrow) adjacent to necrotic areas (star, upper right image). Lower right images shows negative control staining of healthy brain tissue. Scale bar indicates 50 μm.

The presented data suggests that therapeutic interference with RTK-AXL phosphorylation using the selective small molecule inhibitor BMS-777607 is an effective means to interfere with multiple aspects of glioma growth. Although previous reports have shown that RTK-AXL is expressed in GBM tissue (10), it remains unknown whether the RTK-AXL signaling pathway is activated in humans. We therefore established a Western Blot analysis, immunoprecipitation, and immunohistochemical staining for P-AXL for human GBM tissue and used normal brain tissue derived from epilepsy surgery as negative control. We analyzed 16 GBM samples of patients in the age of 44–77 years (mean age at diagnosis: 63 years). 8 of them had newly diagnosed GBM, 8 had first recurrence of their GBM following surgery and standard radio-/chemotherapy, and underwent reoperation. For the first time, these analyses demonstrated that RTK-AXL is abundantly activated in GBM tissue. Western Blot analysis showed strong protein expression as well as phosphorylation of RTK-AXL in both primary and recurrent GBMs with a trend for even higher RTK-AXL expression in recurrent tumors (Figure 6A, upper and lower image). Immunohistochemistry localized P-AXL in tumor cells in hypercellular regions (Figure 6B, upper left image) as well as in glioma blood vessels of primary GBM (Figure 6B, lower left image). Interestingly RTK-AXL was strongest phosphorylated in tumor cells surrounding necrotic zones, so called pseudo-palisades (Figure 6B, upper right image).

## DISCUSSION

The principle findings of our study are that a therapeutic inhibition of RTK-AXL results in a decreased migration and invasion and increase of apoptotic events in glioma cells *in vitro* and *in vivo*. The targeted inhibition of RTK-AXL phosphorylation leads to a significant decrease in tumor volume or even a complete regression of tumor mass in case of SF126 and U118MG xenografts. As previously shown by our group, antitumor effect of RTK-AXL inhibition is related to pro-apoptotic, anti-proliferative and anti-invasive effects in the tumor [15]. Our data confirm the role of RTK-AXL as mediator of resistance to apoptosis via caspase 3 signaling [6, 7]. The up-regulation of P-AXL under starving condition explains the idea of an anti-apoptotic feedback loop mechanism. Another finding of this study is that we detect an effect of RTK-AXL inhibition toward endothelial cells resulting in an increased ability of tube formation *in vitro* and less neovascularization *in vivo*. We therefore conclude that inhibition of RTK-AXL leads to an anti-angiogenic effect and therefore represents another possible point of action in glioma therapy.

The clinical relevance of RTK-AXL has been illuminated by Hutterer et al. [10]. He presented clinical data showing, that RTK-AXL is mainly expressed in pseudo-palisades and GFAP positive tumor cells, whereas the specific ligand of RTK-AXL Gas6 is expressed in hypoxic border zone but not in pseudo-palisades [10]. Our data localizes for the first time areas of phosphorylated RTK-AXL in human GBM tissue. RTK-AXL is phosphorylated in different sides of GBM tissue especially in characteristic lesions of this disease like hypercellular zones, pseudo-palisades, and vascular proliferates. Activation mechanisms of RTK-AXL were not investigated in this study, but further research has to focus on the role of RTK-AXL-specific ligand Gas6 in GBM. Besides activation of RTK-AXL via Gas6 other mechanisms have to be considered like phosphorylation by ROS (reactive oxygen species) and EGFR (epidermal growth factor receptor) interaction [6, 20, 21]. It has been shown that resistance to EGFR monoclonal antibodies is leading to an up-regulation of AXL [22]. The underlying resistance mechanism is associated with morphological changes from epithelial to mesenchymal phenotype (EMT) in the tumor tissue. It has been shown repeatedly that AXL is up-regulated by EMT [23]. For this reason it would be particularly interesting to study a combination therapy with EGFR and RTK-AXL inhibitors to exhibit synergistic effect with respect to tumor control and treatment response.

Due to complexity and heterogeneity of GBMs it is unlikely that single molecular therapy would achieve substantial anti-tumoral effect in GBM patients. But compared to other targets, inhibition of RTK-AXL leads to multiple anti-cancer effects blocking proliferation, invasion, and angiogenesis. This approach displays a clear advantage of the target RTK-AXL in glioma therapy.

Despite the promising results with BMS-777607 treatment in a glioma model we have to state, that in very few cases RTK inhibitors made it to clinical use in GBM treatment and most multi-kinase inhibitors did not fulfill expectations in clinical practice [17, 24]. One point of failure of tyrosine kinase inhibitors in clinical practice is toxicity. In case of sunitinib, clinical trials revealed minimal anti-GBM activity and substantial toxicity [25]. In contrast to this multi-kinase inhibitor, BMS-777607 displays no toxicity defined by weight loss and morbidity at a concentration of 30–50 mg/kg BW in animal models [18, 26]. Detailed information concerning the safety profile and maximum tolerated dose is expected by results of a phase I multiple ascending dose study of BMS-777607 (ClinicalTrials.gov, Identifier: NCT01721148) in December 2016.

Despite toxicity, clinical failure of tyrosine kinase inhibitors in glioma therapy is further associated with kinase switch, mal penetrance of blood brain barrier (BBB) and poor penetrance of tumor tissue. Selectivity of BMS-777607 towards Met kinase superfamily (Ron, AXL, Tyro-3, and Mer) has been shown previously [18]. Our results demonstrate that BMS-777607 is inhibiting RTK-AXL at the administered dosage of 12.5 μM *in vitro* and 30 mg/kg BW *in vivo* without affecting MET kinase phosphorylation. For that reason, we confirm crossing of the BBB and tissue penetrance with the proof of targeted inhibition of RTK-AXL phosphorylation in our xenografts. Furthermore we address effects of BMS-777607 as AXL specific in this study. So far we did not observe kinase switch toward MET kinase activation. Nevertheless even if BMS-777607 might develop anti-MET kinase activity in clinical use, this could lead to a more pronounced antitumor effect knowing of the relevance of Met kinase superfamily in gliomagenesis and glioma progression [27–31]. Yet, research has to focus on resistance mechanisms and suitable drug combinations to overcome escape strategies of these tumors [11, 16]. Further studies are needed to evaluate RTK-AXL inhibition on patient derived glioma xenografts as basis for application in clinical trials.

## MATERIALS AND METHODS

### Compound

Small molecule tyrosine kinase inhibitor BMS-777607 was purchased by ShangHai Biochempartner Co., Limited (Cas No.:1196681-44-3) with a purity > 98%, which passed an independent quality control check by LC/MS and NMR analysis at the Lead Discovery Center GmbH, Dortmund Germany. BMS-777607 (MW: 512.90 g/mol) was recently published as a MET kinase inhibitor (IC_50_ = 3.9 nM), but has been shown to be more selective for AXL (IC_50_ = 1.1 nM) [18]. IC_50_ value of BMS-777607 was determined in MTT assay for each cell assessing at least 8 different concentrations. According to the results, the *in vitro* assay concentration of BMS-777607 was 12.5 μM. The compound was diluted in DMSO (Roth) and DMEM (Gibco). The control group was treated with equal amounts of solvent.

*In vivo* compound was used at a concentration of 30–100 mg/kg of body weight (BW). The compound was diluted in DMSO and PEG300 (Sigma Aldrich). The compound was administered twice a day via intraperitoneal (i.p.) injection. The control group received injections containing the solvent agents.

### Cell culture

Human high-grade glioma cells U118MG were obtained from American Type Culture Collection (ATCC). Human high-grade glioma cells SF126 were obtained from the JCRB Cell Bank. Both were primary tumor cell cultures derived from surgical specimens of human GBM, WHO Grade IV. Cell authentication was carried out with LGC Standards Cell line Authentication service in June 2014 with 16 loci service of short tandem repeat profile. Tumor cells were maintained as monolayer cultured in tumor growth medium at 37°C, 5% CO_2_, 95% humidity in a tissue culture incubator. Growth medium was comprised of Dulbecco's modified Eagle's medium (DMEM, Invitrogen) supplemented with 10% fetal calf serum (FCS) and 1% antibiotics (penicillin/steptamycin). Prior starting experiments, each cell population was grown in equal (80%) confluence in culture dishes under normal conditions or starving conditions (culture medium containing 0% FCS). Cell count and cell vitality was assessed with CASY^®^ Cell Counter TT (OLS).

HUVEC cell line was obtained from PromoCell. Cells were cultured accordingly to manufactures instructions.

### Spheroids

Cells were relabeled prior forming spheroids with DiI (invitrogen) according to manufactures instructions. Cells were seeded in an uncoated, non-adhesive 96-well plate (Sarstedt). Culture medium contained 20% Methocell medium consisting of 6 gr. carboxymethylcellulose (Sigma Aldrich) and 250 ml of the preheated ECBM (Endothial cell growth medium, Lonza). 5.000 cells were plated per well in 100 μl Methocell medium. Spheroids grew within 48 hours. Round-shaped spheroids were selected for experiments with a diameter size of 300–500 μm.

### Cell viability assay-MTT assay

5.000 cells were seeded per well of a 96-well plate in triplicates and incubated with 0.1 ml DMEM supplemented with 5% FCS. Fractions were treated with DMSO or treated with compound at different concentrations (1.56 μM–50 μM). Culture medium was replaced or exchanged after 24, 48 and 72 hours. 100 μl of MTT reagent (Thiazolyl Blue Tetrazolium Bromide, Sigma) was added and incubated for 3 hour. Crystals were solved with 100 μl DMSO-isopropanol (Sigma Aldrich) (1:1) under constant shacking for 10 minutes. Absorption of supernatant was measured at 570 nm with plate reader (infinite200).

### Apoptosis assay

Apoptosis was measured with caspase3/CPP32 Colorimetric Assay Kit (Biovision). Cells were cultured for 24 hours with 50 ng/ml TNF-alpha (Dianova) or with compound at a concentration of 12.5 μM. The non-induced control was treated with solvent DMSO at a final concentration of 0.5% (Sigma Aldrich). 200 μg protein were incubated with 2 × Reaction buffer containing 10 mM DTT and DEVD-pNA substrate. Absorption was determined at 405 nm. Fold increase in CPP32 activity was determined by comparing results of treated group with the level of the non-induced control. Assay was performed in triplicates for the calculation of mean values and standard deviation (*n* = 3).

### Boyden chamber migration assay

Experiments were performed with 50.000 cells per chamber. Prior starting the experiment the inserts were coated with fibronectin (Sigma Aldrich) at a final concentration of 10 μg/ml in cold PBS. Inserts were incubated with fibronectin for 2 hours in the cell incubator. Cell suspension and compound were added to the upper chamber (compound concentration: 12.5 μM). The lower chamber was filled with 110 μl of fibroblast-conditioned medium (FCM) to stimulate cell migration. A filter with 8 μm pore size separated the upper and lower chamber. After a migration time of 3 hours, cells adhering to the bottom side of the filters were fixed with methanol for 5 minutes. The inserts were dried for 5 minutes and then stained with DAPI (Sigma Aldrich, diluted in PBS 1:100). Cells per field were counted at a magnification of 10 fold, 4 fields per filter were averaged (*n* = 5).

### Orthotopic brain slice invasion assay

For preparation of brain *slice cultures*, 3 to 6-days-old mouse pups (C57BL/6NCrl, Charles River) were used. Brain hemispheres were sectioned in 300 μm thick coronary slices (McIllwain tissue chopper). Slices were separated in ice cold dissection medium containing 99 ml MEM and 1 ml glutamine (Gibco) and cultured onto semipermeable membrane of inserts (Transparent PET membrane, 1.6 × 10^6^-pores/cm^2^, BD, Falcon^®^) with 1 ml culture medium containing 23 ml MEM (Gibco21575), 12.5 ml Horse serum, 12.5 ml BME (Gibco 41010), 1.5 ml of 20% glucose (Roth) and 0.5 ml Glutamine (Life Technologies, L-Glutamine 200 mM). Medium was replaced every two days. 24 hours after cultivation pre-labeled spheroids were placed in the area between striatum and corpus callosum. Invasion of DiI-labeled cells were monitored using confocal microscopy. After 8 days slices were fixed in 4% PFA for 2 hours at 37°C. Nuclear staining of slices was performed with To-PRO-3 according to manufacturer's instructions (Life Technologies). Slices were transferred onto glass slides (Langenbrinck) and mounted in mounting medium (Immuno mount, Thermo Scientific) for confocal analysis. Confocal analysis was performed with Zeiss Confocal microscope. For quantitative analysis distance of invaded cells from border of spheroid was measured (*n* = 8).

### Tube formation assay

10 μl BD matrigel basement membrane matrix (BD 354230) was pipetted to a 15-well plate (μ-Slide Angiogenesis, ibiTreat), matigel solution formed gel within 30 minutes incubation time at 37°C. Harvested HUVEC cells were suspended in a concentration of 200 000 cells/ml with 12.5 μM concentration of compound or vehicle (0.5% DMSO). 50 μl of cell suspension pro well was added onto solidified gel and incubated for 4 to 20 hours. Tube formation was analyzed with Zeiss Axiovision fluorescence microscope. Tube length and branching points were quantified using online service Wimasis Image Analysis (*n* = 5).

### Experimental tumor models

Athymic CD-1 Nu/Nu nude mice (female, obtained from Charles River) were maintained within a pathogen germ-free environment and were used at 6–10 weeks of age. The weight ranges was in a range of 20 to 28 gram. Experiments were performed in accordance with the approved institutional protocol and the guidelines of the Institutional Animal Care and Use Committee. General anesthesia was induced with 7% Ketaminhydrochlorid (Ketavet, Pfizer), 8% Xylaxine (Rompun 2%, Bayer) dissolved in aqua via i.p. injection. Phenoxymethylpenicillin (InfectoCilin, 5 Mega) was administered via intramuscular injection. For stereotactic tumor cell implantation, mice were positioned in the stereotactic platform. A longitudinal skin incision was performed in the mid scalp extending from the ears caudally. The burr hole was drilled with a syringe of 23′G 2 mm posterior and 1.5 mm laterally to bregma. Hamilton syringe was loaded with glioma cells dissolved in DMEM without FCS. Hamilton syringe was inserted gently to a depth of 4 mm. After injection wound was closed with suture (Prolene 2.0, Ethicon). Finally, mice were placed on a heating plate set to 37°C until regaining consciousness. Drinking water was enriched with tramadol calculating 15 mg/kg BW (Grünenthal). Contrast enhanced MRI (gadopentetate dimeglumine; Magnesvist, Bayer) scans were performed under anesthesia with 7T Bruker MRI (PharmaScan 70/16 US, Bruker Software Paravision 5.1). Tumor volumetric analysis was carried out with Analyze 10.0 and ImageJ software. Mice were sacrificed at day 14 and the body was perfused with PBS under anesthesia. The whole brain or tumor was dissected and immediately frozen in liquid nitrogen and stored at −80°C (*n* = 7).

### Immunoprecipitation (IP)

IP was performed using Protein A-Sepherose 4A beads (Life Technologies). Low salt IP-lysis buffer was enriched with Halt^™^ Phosphatase Inhibitor Single-Use Cocktail (Thermo Scientific) and Halt Protease Inhibitor Single-Use Cocktail (Thermo Scientific). Immuncomplexes were prepared with 200 μg protein and 1:50 dilution of anti-AXL (C89E7) rabbit antibody (Cell Signaling). Assay was performed according to previous protocol (14). Detection of RTK-AXL phosphorylation was determined using antibody 4G10 mouse anti-tyrosine antibody (Max Planck Institute of Biochemistry, Dilution 1:2.000).

### Western blotting (WB)

Protein samples were boiled for 5 min with Laemmli sample buffer (Bio-Rad Laboratories) and loaded into pre-poured Tris-HCl-glycine SDS-PAGE gels (stacking gel 4%, resolving gel 6% or 8%). Gels run at 150 V for 1.5 hours following transfer to a polyvinylidene difluoride membrane (PVDF, Bio-Rad Laboratories) at 40 mA constant current for 2 h. Blots were blocked with 5% BSA in 1xTBST, primary and secondary antibodies were dissolved in TBST. Following primary antibodies were used. R & D Systems: human phopho-AXL (Y779) mAb (Clone 713610), Cell Signaling: anti-AXL C89E7 rabbit mAb, phospho-MET (Tyr1234/1235) rabbit mAb (D26) XP^®^. Santa Cruz: Gas6 antibody (C-20). Sigma Aldrich: mouse monoclonal β-actin antibody (clone 1A4). Immunocomplexes were visualized using a second HRP-conjugated anti-rabbit or anti-mouse antibody (Pierce Biotechnology). For development we used ECL Kit (Sigma). ImageJ Software was used for densitometry analysis. Reported values were first normalized to the loading control and then multiplied by a constant to reach the lowest whole integral. Each western blot was carried out with a negative control consistent of sample diluent or beads incubated with antibody and sample diluent only.

### Immunohistochemistry and immunofluorescence cells

Adherent cells were plated onto cover slips and fixed with 4% PFA for 10 minutes at room temperature. Cells were blocked with 1% casein for 45 minutes at room temperature. Antibodies were diluted in 0.5% casein. The primary antibody was incubated for one to two hours, the secondary antibody for one hour. Wash steps were carried out with TBST. Negative control was carried out without primary antibody incubation and is shown in Supplementary Figure S1 for anti-AXL antibody and anti-phospho-AXL antibody.

For immunofluorescence human phopho-AXL (Y779) mAb (Clone 713610, R & D Systems) and anti-AXL C89E7 rabbit mAb (Cell Signaling) were used. The following secondary, fluorescently labeled antibodies were used: FITC-conjugated donkey anti-rabbit IgG (Dianova, 711-095-152), Cy3-conjugated donkey anti-mouse IgG (Dianova, 115-165-146).

### Mouse tissue

Mouse brain was mounted in paraffin block in a.p. orientation. 8–20 μm coronary sections were prepared with cryostat (Microm Cryo-Star HM 560 Cryostat, GMI). Sections were fixed with PFA 4%. Staining of intratumoral vessels and proliferation was carried out with purified rat anti-mouse CD31 antibody (clone MEC13.3, BD Pharmingen^™^) and rabbit anti-Ki-67 antigen monoclonal antibody (clone SP6, Diganostic BioSystems). The following secondary, fluorescently labeled antibodies were used: FITC-conjugated donkey anti-rabbit IgG (Dianova, 711-095-152), Cy3-conjugated donkey anti-rat IgG (Dianova, 712-165-153). Apoptotic activity within the tumor was assessed using Apoptaq kit (Millipore). Staining procedure was done according to manufacturer's instructions.

Tissue staining for AXL and phospho-AXL was carried out on PFA fixed slices. Cells and brain slices were finally counterstained with DAPI (Thermo Scientific). Slices were analyzed using a fluorescence microscope (Zeiss).

For statistical analysis we used ImageJ software. For qualitative analysis of fluorescent signal, all parameters were maintained as set for the initial image.

### Human tissue

Immunohistochemical staining of formalin-fixed, paraffin-embedded (FFPE) tissue sections (4 μm-thick) was performed on a VENTANA Benchmark XT automated staining instrument according to the manufacturer's instructions. Slides were de-paraffinized using EZ prep solution (Ventana Medical Systems, Tucson, AZ) for 30 minutes at 75°C. Antigen retrieval was accomplished on the automated stainer using CC1 solution (Ventana Medical Systems, Tucson, AZ) for 60 minutes at 95°C. Briefly, the anti-phospho-AXL antibody (Human Phospho-AXL (Y779) monoclonal mouse IgG Clone 713610, R & D Systems, dilution 1:50) was applied and developed using the iVIEW DAB Detection Kit (Ventana Medical Systems). All slides were then counterstained with hematoxylin for 4 minutes.

### Patient data

Clinical data was assessed under an institutional review board-approved protocol and de-identified for patient confidentiality. We included 16 GBM patients, which have been treated in our institution in the year 2015. GBM diagnosis was assured of two independent neuropathologists. RTK-AXL and phospho-AXL expression was quantified with IP and WB analysis in 8 patients with primary GBM and in 8 patients with recurrent disease, which received re-resection. FFPE tissue sections of these individual 16 patients were stained with anti-phospho-AXL antibody.

### Microscopy

Images were recorded by fluorescent microscope from Zeiss (Obeserver Z1). Following objectives were used: 5× EC PlnN, 5×/0.16 DIC0 (resolution: 2.0 μm), 10× Pln Apo, 10x/0.45 DIC II (resolution: 0.74 μm), 20× Pln Apo, 20×/0.8 DIC II (resolution: 0.42 μm). We use a HAL 100 and detectors for DAPI, GFP and DSRed. Pictures were processed and recorded with Image software Axio Vision Rel. 4.8. Statistical analysis was carried out with ImageJ Software 1.46r.

### Statistical analysis

Each group consisted of at least triplicates. *In vivo* experiments were performed with five or more animals per group. Animals were de-identified for blinded analysis of MRI volumetry and IHC analysis. Statistical analysis was performed using GraphPad Prism, Version 5.0c. For statistical test we used Student's *T*-Test and one-way ANOVA combined with Bonferroni's multiple comparison test. Significance level was set at alpha = 0.05 (95% confidence intervals). The level of significance was set at *p < 0.05.

## SUPPLEMENTARY MATERIALS FIGURES


