# Supplementary material for: Inhibiting receptor tyrosine kinase AXL with small molecule inhibitor BMS-777607 reduces glioblastoma growth, migration, and invasion in vitro and in vivo
Source: Oncotarget. 2016 Feb 2;7(9):9876–89. doi: 10.18632/oncotarget.7130 (PMC4891090; doi:10.18632/oncotarget.7130)
Supplement: Supplementary file 1 [file oncotarget-07-09876-s001.pdf]

Inhibiting receptor tyrosine kinase AXL with small molecule inhibitor BMS-777607 reduces glioblastoma growth, migration, and invasion *in vitro* and *in vivo*

Supplementary Materials

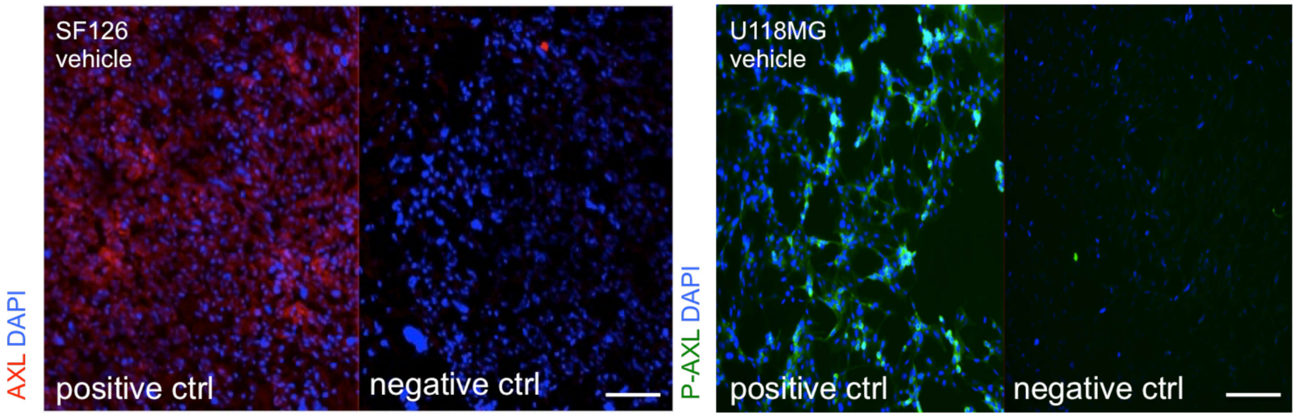

Supplementary Figure S1: Negative and positive control with incubation of secondary antibody only in case of anti-AXL staining (left image) and anti-phospho-AXL staining (right image).

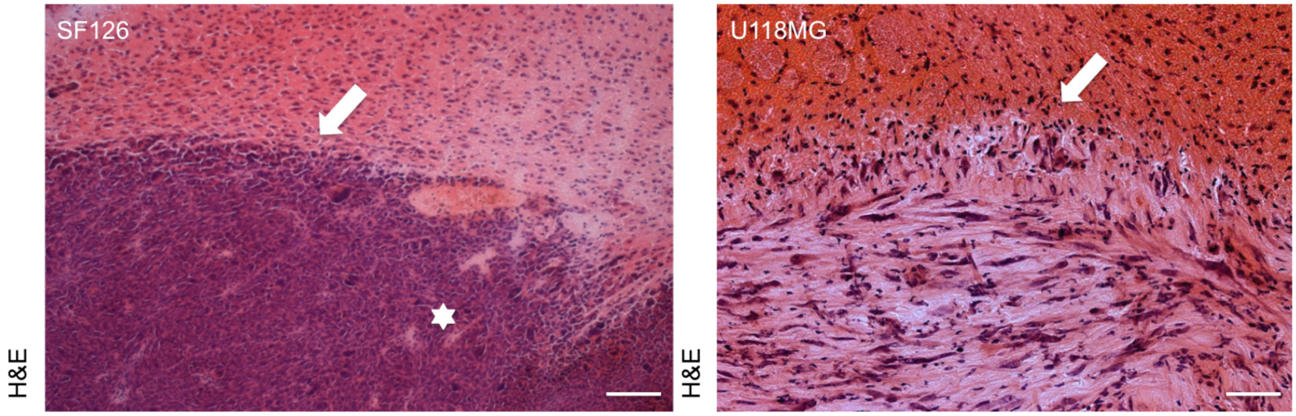

Supplementary Figure S2: H & E staining displays different growth pattern of both cells. The SF126 tumor (left image, arrow) show vascular proliferates (left image, star) and less invasive growth compared to U118MG (right image, arrow).

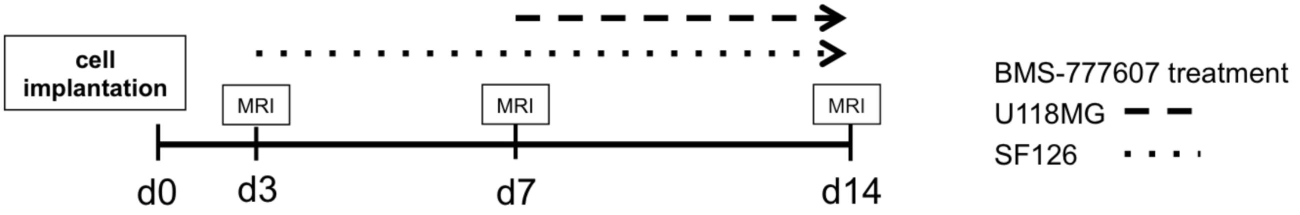

Supplementary Figure S3: Experimental setting of intracranial xenograft model with U118MG and SF126 cells. Treatment started after proven tumor mass on MRI.
